# Navigating the Diagnostic Challenges of Mycoplasma and Legionella Coinfection Through Serological Testing

**DOI:** 10.7759/cureus.56691

**Published:** 2024-03-22

**Authors:** Sushma Edara, Sivaprasad Nalluri

**Affiliations:** 1 Internal Medicine, Interfaith Medical Center, New York, USA; 2 Internal Medicine, South Dayton Acute Care Consultants, Dayton, USA

**Keywords:** co-infection, bacterial coinfection, atypical pathogen, urine antigen testing, serology, legionella pneumophila, mycoplasma pneumonia

## Abstract

Diagnosing community-acquired pneumonia (CAP) is increasingly challenging, especially with the emergence of atypical pathogens such as *Mycoplasma pneumoniae *and* Legionella pneumophila*. This report presents the case of a 60-year-old male exhibiting lethargy and decreased oral intake, with a medical history marked by chronic kidney disease and benign prostate hyperplasia. Despite a positive *Legionella* urine antigen, the clinical and radiological profile did not align with *Legionella* pneumonia. Elevated *M. pneumoniae* IgM antibody titers further complicated the diagnostic scenario. We explore the complexities of distinguishing coinfection from primary infection, highlight the limitations of serological testing, and promote a comprehensive diagnostic strategy customized to individual patient circumstances. This case emphasizes the importance of comprehensive assessment strategies to understand atypical pneumonia presentations, particularly within complex clinical scenarios.

## Introduction

Diagnosing respiratory infections caused by atypical pathogens such as *Chalmydia pneumoniae*, *Mycoplasma pneumoniae*, and* Legionella pneumophila* presents challenges, particularly in distinguishing between coinfections and genuine infections. This case highlights a 60-year-old male with a complex medical history who presented with lethargy and decreased oral intake. Despite atypical clinical findings such as lethargy and radiological results, he was ultimately diagnosed with a coinfection of *M. pneumoniae* and *L. pneumophila*. Colonization by atypical organisms may yield positive results in serological testing, indicating exposure to the pathogen without necessarily confirming active infection. We want to highlight that a positive IgM for *Mycoplasma* and a urine antigen for *Legionella* do not definitively indicate coinfection. Depending exclusively on serological testing reveals its limitations in sensitivity and specificity, making it challenging to differentiate between confection and genuine infection caused by atypical pathogens, thereby adding to the complexity of diagnosis.

## Case presentation

**Figure 1:**
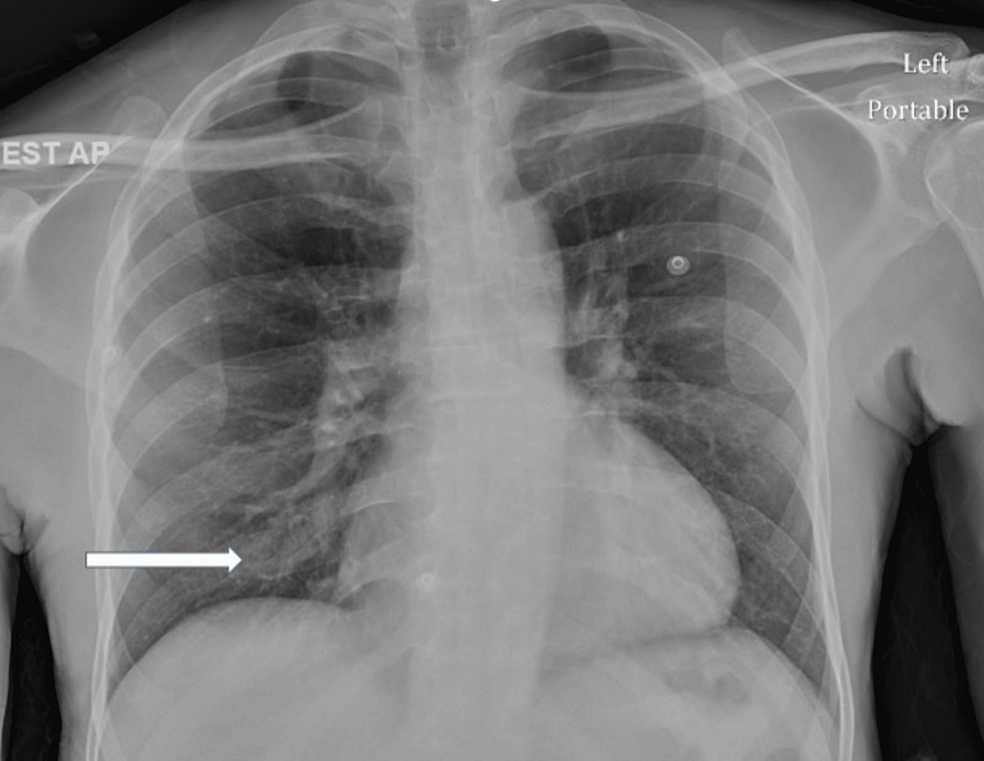
Chest X-ray showing right lower lobe infiltrates (arrow)

A 60-year-old male was admitted due to a week-long history of lethargy and decreased oral intake. His medical background included hypertension, prediabetes, stage 5 chronic kidney disease, chronic alcohol abuse, and benign prostatic hyperplasia with bilateral hydroureteronephrosis. He denied having a cough, shortness of breath, or chest discomfort. He had no history of recent travel. Despite being afebrile, his blood pressure was 132/72 mmHg, his heart rate was 67 beats per minute, and his respiratory rate was 16 breaths per minute, with oxygen saturation at 97% on room air. A chest X-ray revealed infiltrates in the right lower lobe (Figure 1), while a physical examination indicated bilateral vesicular breath sounds. Laboratory tests showed no leukocytosis and normal electrolytes but elevated blood urea nitrogen (BUN) and creatinine levels. Further evaluation yielded negative blood cultures, along with elevated *M. pneumoniae* IgM (1092) and IgG (543), antibody titers, and a positive urine antigen for *L. pneumophila* serogroup 1. *Chlamydia pneumoniae* polymerase chain reaction (PCR), methicillin-resistant *Staphylococcus aureus* (MRSA) PCR, flu, COVID-19, and respiratory syncytial virus (RSV) were negative. Consequently, the patient was treated with azithromycin for seven days based on these laboratory results.

## Discussion

Atypical pathogens such as *M. pneumoniae* and *L. pneumophila* are increasingly recognized as common causes of community-acquired pneumonia (CAP) [1], highlighting the need to accurately identify the pathogens responsible. Coinfections can further complicate the clinical presentation, necessitating comprehensive testing and tailored treatment strategies.

*Mycoplasma*, characterized by its gram-negative bacteria lacking a cell wall, is known to cause respiratory infections termed '*Mycoplasma* pneumonia' or 'walking pneumonia' (a milder variant of pneumonia). Common symptoms include cough, fever, sore throat, and chest discomfort, with infections often persisting in the population, leading to prolonged symptoms. In elderly or immunocompromised individuals, it may progress to severe pneumonia, occasionally accompanied by neurological complications [2]. Diagnosis typically involves direct PCR or nucleic acid amplification tests (NAATs) due to their high sensitivity and specificity compared to serology and culture [3]. When there's a strong clinical suspicion of *Mycoplasma* pneumonia, direct PCR or NAATs are typically utilized for diagnosis, offering superior sensitivity and specificity compared to serology and culture. However, NAATs cannot discern between an active infection, coinfection, or asymptomatic carriage. In the absence of molecular testing, serology serves as an alternative. The gold standard for serological diagnosis involves detecting a fourfold rise in IgG titers between acute and convalescent samples, typically taken around four weeks apart, but this approach is often impractical [3]. A single elevated IgM titer is used for a presumptive alternate diagnosis, despite its relatively low specificity. Combining IgM titer results with NAAT or direct PCR can aid in confirming the diagnosis [3]. Cultures are not beneficial for *Mycoplasma* due to the absence of a cell wall, requiring specialized media, and potentially taking two to three weeks for growth.

*Legionella*, another genus of gram-negative bacteria, can cause severe respiratory infections known as 'Legionnaires disease*'* or a milder illness called 'Pontiac fever*'*. These bacteria are commonly found in freshwater environments like lakes and rivers and can proliferate in human-made water systems such as air conditioning systems, hot tubs, and plumbing. Legionnaires disease manifests with symptoms like high fever, cough, shortness of breath, muscle aches, and sometimes gastrointestinal symptoms. Pontiac fever, on the other hand, is a less severe illness resembling flu-like symptoms. Individuals with weakened immune systems and underlying comorbid conditions are particularly vulnerable to these infections. Within the *Legionella* genus, multiple species and serotypes exist, allowing for the differentiation of various strains or subtypes of the bacteria. Key testing options for *Legionella* infection include urine antigen testing, PCR from sputum samples, and culture, as shown in Table 1 [4]. Polymerase chain reaction offers high diagnostic accuracy and detects all *Legionella* species and serogroups in patients with pneumonia. In regions with a high prevalence of the* L. pneumophila* serogroup, when PCR is unavailable or sputum cannot be obtained, urine antigen testing serves as an alternative. However, it only detects *L. pneumophila* serotype 1 [5], and while advantageous for its rapid turnaround time and high specificity, a negative result from a urine antigen assay does not rule out infection.

**Table 1:** Sensitivity and specificity of diagnostic test for Legionella species PCR: Polymerase chain reaction

Test characteristic	Urine antigen	PCR	Culture
Strains detected	Detects on *Legionella* ​​​​*pneumophila* serogroups	All species and serogroups	All species and serogroups
Time to results	<1 hour	Hours	>3 days
Overall sensitivity	70%-80 %	Exceeds culture	<10% to 80%
Overall specificity	>99%	>99%	100%

There have been limited studies in the past examining coinfections versus cross-reactivity between *M. pneumoniae* and* L. pneumophila* [6]. The differentiation between a genuine infection and a coinfection poses a significant challenge, especially when diagnosis relies heavily on serological testing, as noted in Saladi et al. It is important to emphasize that a positive IgM result or urine antigen detection for *Legionella* alone does not automatically imply a coinfection. This study highlights the inadequacies of relying solely on serological testing, showcasing poor sensitivity and specificity, as discussed earlier.

## Conclusions

Differentiating between a coinfection and a genuine infection involving atypical respiratory pathogens has proven to be a complex task. To improve diagnostic accuracy, incorporating *Legionella *PCR for *L. pneumophila* and incorporating *M. pneumoniae* serology alongside NAAT or PCR can provide valuable additional evidence. It is imperative to conduct vigilant surveillance of respiratory pathogens, encouraging physicians to adopt a proactive stance when diagnosing atypical pathogens.
